# Elimination of quiescent slow-cycling cells via reducing quiescence depth by natural compounds purified from *Ganoderma lucidum*

**DOI:** 10.18632/oncotarget.14634

**Published:** 2017-01-13

**Authors:** Jian Dai, Matthew A. Miller, Nicholas J. Everetts, Xia Wang, Peng Li, Ye Li, Jian-Hua Xu, Guang Yao

**Affiliations:** ^1^ Department of Molecular and Cellular Biology, University of Arizona, Tucson, AZ, USA; ^2^ School of Pharmacy and Fujian Key Laboratory of Natural Medicine Pharmacology, Fujian Medical University, Fuzhou, Fujian, China; ^3^ Fujian Xianzhilou Biological Science and Technology Co, Ltd, Fuzhou, Fujian, China; ^4^ Jiangsu Academy of Agricultural sciences, Nanjing, Jiangsu, China; ^5^ Arizona Cancer Center, University of Arizona, Tucson, AZ, USA

**Keywords:** quiescence, natural compounds, ganoderma lucidum, slow-cycling cells, cancer stem cells

## Abstract

The medical mushroom *Ganoderma lucidum* has long been used in traditional Chinese medicine and shown effective in the treatment of many diseases including cancer. Here we studied the cytotoxic effects of two natural compounds purified from *Ganoderma lucidum*, ergosterol peroxide and ganodermanondiol. We found that these two compounds exhibited cytotoxicity not only against fast proliferating cells, but on quiescent, slow-cycling cells. Using a fibroblast cell-quiescence model, we found that the cytotoxicity on quiescent cells was due to induced apoptosis, and was associated with a shallower quiescent state in compound-treated cells, resultant from the increased basal activity of an Rb-E2F bistable switch that controls quiescence exit. Accordingly, we showed that quiescent breast cancer cells (MCF7), compared to its non-transformed counterpart (MCF10A), were preferentially killed by ergosterol peroxide and ganodermanondiol treatment presumably due to their already less stable quiescent state. The cytotoxic effect of natural *Ganoderma lucidum* compounds against quiescent cells, preferentially on quiescent cancer cells vs. non-cancer cells, may help future antitumor development against the slow-cycling cancer cell subpopulations including cancer stem and progenitor cells.

## INTRODUCTION

*Ganoderma lucidum*, also famously known in Asian countries as *Lingzhi* or *Reishi*, is a mushroom species used in traditional Chinese medicine for disease treatment and longevity promotion for more than 2,000 years. The fruiting body and spores of *Ganoderma lucidum* contain hundreds or more of bioactive compounds primarily belonging to categories of triterpenoids (ganoderic acid), polysaccharides, and steroids [1–3]. It has been widely reported that the active components of *Ganoderma lucidum* exhibit therapeutic antitumor, antiviral, anti-inflammatory and immunomodulatory effects, and are beneficial to treatment of diseases including cancer, AIDS, hypertension, hepatitis, and diabetes [4–8].

The antitumor effects of *Ganoderma lucidum* have been linked to cell cycle arrest, induction of cytotoxicity and apoptosis, induction of differentiation, suppression of angiogenesis and cell migration, and immunomodulation [9–12]. These documented effects primarily regard proliferating cancer cells. Little is known about the effects of *Ganoderma lucidum* against the quiescent, slow-cycling subpopulation of cancer cells (including but not limited to cancer stem cells), which often leads to cancer recurrence [13, 14].

In this study, we tested whether natural compounds from *Ganoderma lucidum* have inhibitory and cytotoxic effects on quiescent, slow-cycling cells. To this end, we started with four natural compounds (ergosterol, ganodermanontriol, ergosterol peroxide, and ganodermanondiol) that have been shown to exert potent cytotoxicity against proliferating and aggressive cancer cells [10, 15–20], and can be purified to high quality and sufficient quantity from *Ganoderma lucidum* using our previously established methods [19, 20]. Two of the four compounds, ergosterol peroxide and ganodermanondiol, were found to exhibit significant cytotoxicity against quiescent cells in our pilot test, and thus selected for further investigation in this work.

Here we report that ergosterol peroxide and ganodermanondiol, which belong to triterpenoid and steroid categories, respectively, exhibited potent cytotoxic and apoptotic effects in a fibroblast cell-quiescence model under two quiescence-inducing signals, serum starvation and cell contact inhibition. We found that the cytotoxicity in quiescent fibroblasts was associated with the reduction of quiescence depth as indicated by the increased basal activity of the Rb-E2F bistable switch [21–23]. Since quiescence provides a protection against cellular stress and toxicity [24, 25], the shallowing of the quiescence state led to the sensitization of cells to quiescence exit and apoptosis.

We further tested whether quiescent, slow-cycling cancer cells, presumably already at a less stable and shallower quiescent state compared to normal quiescent cells, are more sensitive to ergosterol peroxide and ganodermanondiol treatment. In this regard, we compared MCF7 breast cancer cells and its non-transformed counterpart MCF10A breast epithelial cells that were both induced to quiescence by serum starvation. We found that ergosterol peroxide and ganodermanondiol induced stronger cytotoxicity in quiescent MCF7 vs. MCF10A cells. This effect of natural *Ganoderma lucidum* compounds to target quiescent slow-cycling cancer cells may help future development of novel chemotherapeutic agents against cancer stem and progenitor cells for the prevention of cancer recurrence.

## RESULTS

### Ergosterol peroxide and ganodermanondiol induced cytotoxicity in proliferating cells

**Figure 1:**
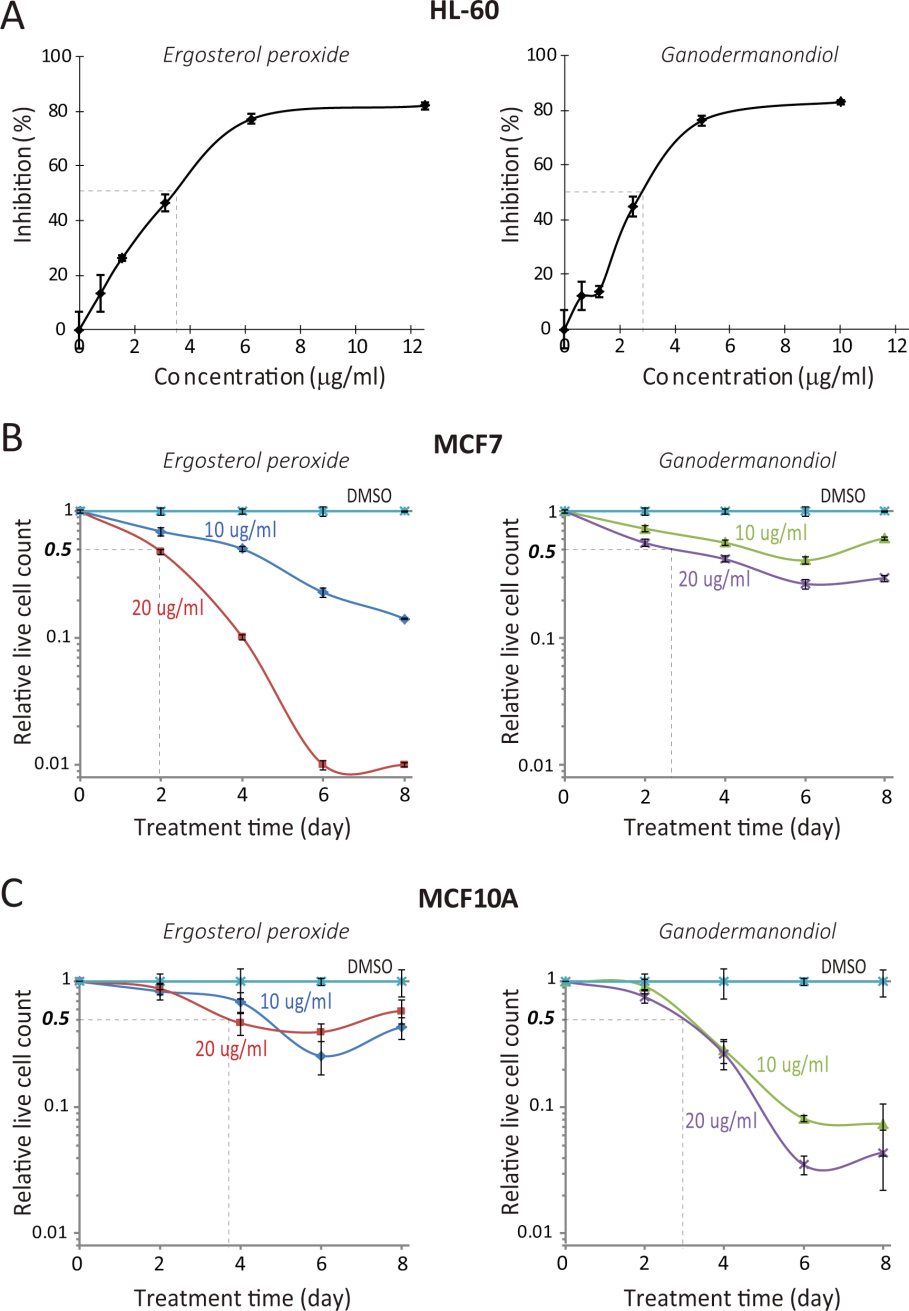
Compound cytotoxicity against proliferating cells (A) Compound inhibition effect on cycling HL-60 lymphoma cells. HL-60 cells were treated with ergosterol peroxide or ganodermanondiol at indicated concentrations (x-axis) for 48 hours. The inhibition rate (y-axis) of compound was determined as (Mc-Mt)/Mc*100%, where Mt and Mc corresponded to the average readings of treated samples and non-treated controls (triplicates) in the MTS assay, respectively. Error bar indicates the standard error of the mean. (B, C) Compound cytotoxicity time course in cycling MCF7 and MCF10A cells. MCF7 cells (B) and MCF10A cells (C) were treated with ergosterol peroxide or ganodermanondiol at indicated concentrations. The relative live cell count (y-axis) refers to the ratio of the median number of live cells in a compound-treated sample over that in the DMSO vehicle control (four replicates each). Live cell count was determined by the PI fluorescence assay (see Methods) at the indicated day (x-axis) during the course of treatment. Error bar indicates the standard error of the median (of four replicates).

Using our previously established methods [19, 20], we isolated and purified ergosterol peroxide and ganodermanondiol (see Table 1 for structure) from the fruiting body of *Ganoderma lucidum* (see Methods). Consistent with earlier reports [10, 15–20], we found that ergosterol peroxide and ganodermanondiol exhibited cytotoxicity against proliferating cancer cells. With HL-60 lymphoma cells, the half lethal concentrations (i.e., required to kill 50% of the cell population, LC50s) were 3.5 and 2.9 μg/ml, respectively, with ergosterol peroxide and ganodermanondiol treatment for 2 days (Figure 1A). With MCF7 breast cancer epithelial cells, cytotoxicity was seen at higher compound doses and longer treatment durations: LC50s were estimated at 20 μg/ml with ergosterol peroxide and ganodermanondiol treatment for about 2 and 2.6 days, respectively (Figure 1B). Ergosterol peroxide and ganodermanondiol also induced cytotoxicity in proliferating non-cancer cells. With MCF10A normal human breast epithelial cells, LC50s were estimated at 20 μg/ml with ergosterol peroxide and ganodermanondiol treatment for about 3.7 and 3 days, respectively (Figure 1C), which were closer to the LC50s of these compounds in treating MCF7 cells compared to treating HL-60 cells.

**Table 1:** Structure of ergosterol peroxide and ganodermanondiol

Name	Molecular formula	Structure	Evaluation method
Ergosterol peroxide	C_28_H_44_O_3_	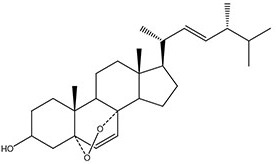	^1^H-NMR ^13^C-NMR
Ganodermanondiol	C_30_H_48_O_3_	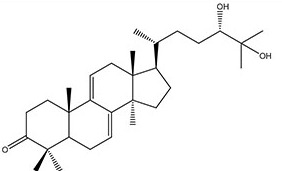	ESI-MS ^1^H-NMR ^13^C-NMR

### Ergosterol peroxide and ganodermanondiol induced cytotoxicity and apoptosis in a fibroblast cell-quiescence model

**Figure 2:**
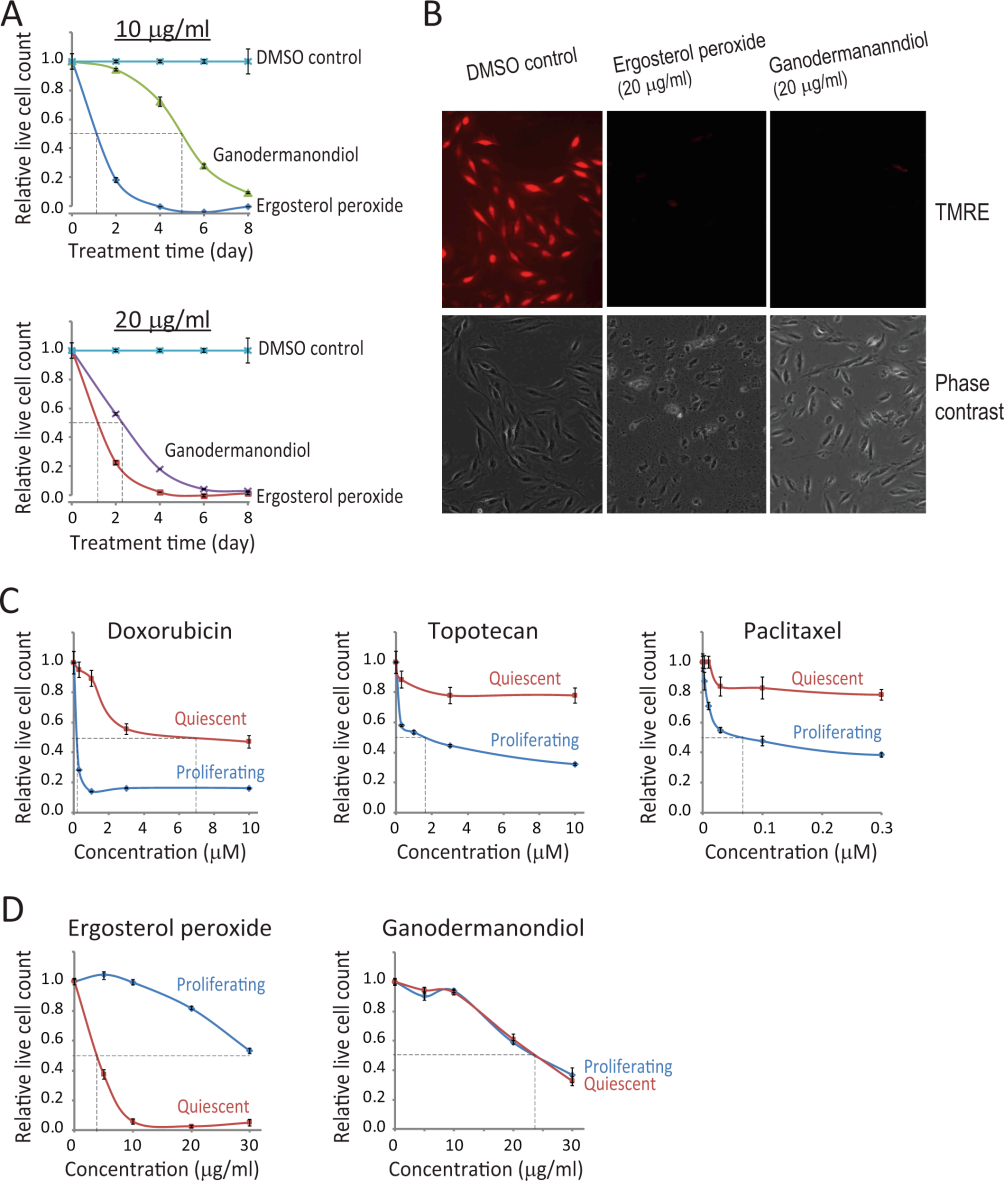
Compound cytotoxicity in a fibroblast quiescence model by inducing apoptosis (A) Compound cytotoxicity time course in quiescent REF/E23 cells. Serum starvation-induced quiescent REF/E23 cells were treated with ergosterol peroxide or ganodermanondiol at 10 and 20 μg/ml as indicated. The relative live cell count (y-axis) was determined as in Figure 1B at the indicated day (x-axis) during the course of treatment. Error bar indicates the standard error of the median (of four replicates). (B) Induced apoptosis. Serum starvation-induced quiescent REF/E23 cells were treated with ergosterol peroxide or ganodermanondiol at 20 μg/ml or DMSO vehicle control. Cells were assayed for mitochondrial membrane potential using TMRE staining after 1 day of treatment. Phase-contrast images were acquired from the same microscopy fields of the corresponding TMRE images. (C, D) Cytotoxicity in proliferating vs. quiescent cells. Actively cycling or serum starvation-induced quiescent REF/E23 cells were treated with doxorubicin, topotecan, and paclitaxel (C) or ergosterol peroxide and ganodermanondiol (D) at indicated concentrations for 48 hours (concentration = 0: DMSO vehicle control). The relative live cell count (y-axis) was subsequently determined as in Figure 1B. Error bar indicates the standard error of the median (of four replicates).

Next, we tested the cytotoxicity of ergosterol peroxide and ganodermanondiol in quiescent cells. Previously, from a rat embryonic fibroblast REF52 cell line we have derived a cell-quiescence model (REF/E23 cell clone), and with which we demonstrated the control of the quiescence-to-proliferation transition by an Rb-E2F bistable switch [21]. Here we tested the effects of ergosterol peroxide and ganodermanondiol using the REF/E23 cell-quiescence model. REF/E23 cells were induced to quiescence by serum starvation for 2 days, and then subjected to ergosterol peroxide and ganodermanondiol treatments for up to 8 days. We found that both ergosterol peroxide and ganodermanondiol caused cytotoxicity in quiescent REF/E23 cells. Ergosterol peroxide treatment at 10 μg/ml for ~1 day caused a 50% reduction in the live cell count compared to that in the DMSO control (i.e., relative live cell count = 0.5, Figure 2A), while similar effects were seen with ganodermanondiol treatment at 20 μg/ml for ~2 days (Figure 2A).

Further investigation suggested that the cytotoxicity caused by ergosterol peroxide and ganodermanondiol in quiescent REF/E23 cells was due to apoptosis induction. Different from cells under the DMSO vehicle control, cells treated with ergosterol peroxide and ganodermanondiol displayed the characteristic loss of mitochondrial membrane potential (TMRE staining, Figure 2B), as well as externalization of inner membrane phospholipids to cell surface (Annexin V staining, Supplementary Figure 1A) and nuclei condensation and fragmentation (Hoechst dye staining, Supplementary Figure 1B).

We also examined the effects of ergosterol peroxide and ganodermanondiol on REF/E23 cells induced to quiescence by cell contact inhibition, another quiescence inducing condition. Under contact inhibition, we found that quiescent REF/E23 cells treated with ergosterol peroxide underwent apoptosis, as seen by their loss of mitochondrial membrane potential compared to DMSO treated cells (Supplementary Figure 2A). Contact inhibited quiescent REF/E23 cells treated with ganodermanondiol also exhibited noticeable loss of mitochondrial membrane potential, although to a lesser degree compared to that in ergosterol peroxide treatment (Supplementary Figure 2A). Consistently, we observed that over the 8-day treatment of contact inhibited REF/E23 cells with ergosterol peroxide (but not with ganodermanondiol or DMSO), certain areas appeared on the culture plate in which cell size increased or even empty space emerged, indicating a decrease of cell number due to cell death (Supplementary Figure 2B).

### Ergosterol peroxide and ganodermanondiol are much more effective in targeting quiescent cells than chemotherapy drugs in the fibroblast cell-quiescence model

Our tests above indicated that ergosterol peroxide and ganodermanondiol exhibited cytotoxicity against both proliferating and quiescent cells. Next, we further compared these two compounds with three widely used chemotherapy drugs (doxorubicin, paclitaxel, and topotecan) in their cytotoxic effects in the REF/E23 cell model.

We found that doxorubicin, paclitaxel, and topotecan preferentially kill proliferating vs. quiescent cells as expected. As seen in Figure 2C, the LC50 of doxorubicin, paclitaxel, and topotecan treatment for two days in proliferating cells (LC50_p_) is at least 15–30 fold smaller than that in quiescent cells (LC50_q_)–doxorubicin: LC50_p_ < 0.3 μM, LC50_q_ = 7 μM; topotecan: LC50_p_ < 2 μM, LC50_q_ > 30 μM (Supplementary Figure 3A); paclitaxel: LC50_p_ < 0.1 μM, LC50_q_> 3 μM (Supplementary Figure 3B). In contrast, as seen in Figure 2D, ergosterol peroxide exhibited stronger cytotoxicity in quiescent vs. proliferating cells (LC50_p_ ≥ 30 μg/ml, LC50_q_ < 5 μg/ml), and ganodermanondiol exhibited the same effectiveness in targeting both quiescent and proliferating cells (LC50_p_ = LC50_q_ = 24 μg/ml). These results suggest that ergosterol peroxide and ganodermanondiol are much more effective in targeting quiescent REF/E23 cells compared to chemotherapy drugs doxorubicin, topotecan and paclitaxel.

### Ergosterol peroxide and ganodermanondiol reduced quiescence depth and led to shallow quiescence

Next, we studied potential mechanisms underlying the cytotoxic effects of ergosterol peroxide and ganodermanondiol in quiescent cells. Cellular quiescence is often referred to as the G0 phase outside of the proliferative cell cycle. It is in fact not a single homogeneous state but with varying depth [26–29]. Compared to deep quiescent cells, shallow quiescent cells are more sensitive to growth signals and are primed to proliferation [27–31]. It has been well documented that the quiescence-to-proliferation transition is controlled by the Rb-E2F pathway [32–35]. E2F transcriptional activators (E2F1-3a, E2F for short) are among the few genes that are both necessary and sufficient to drive quiescence exit [36, 37]. Previously we have further demonstrated that the Rb-E2F pathway functions as a bistable switch to generate an all-or-none E2F activation, which underlies the all-or-none transition between quiescence and proliferation [21]. We also showed that the serum threshold to activate the Rb-E2F bistable switch can serve as an indicator of quiescence depth [23].

**Figure 3:**
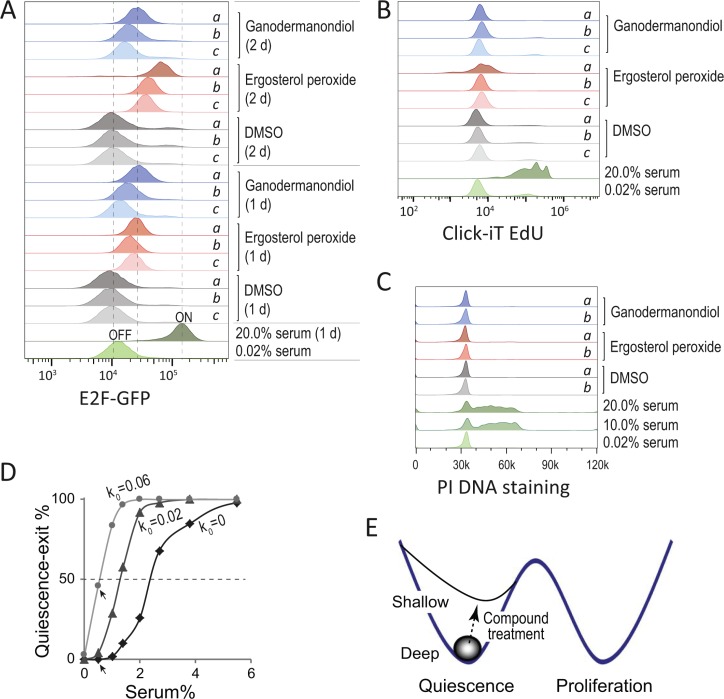
Compound treatment reduces quiescence depth (A–C) Treated cells remained quiescent but at a shallower state with increased E2F expression. REF/E23 cells were induced to quiescence by serum starvation (0.02% serum) for 2 days, and subsequently treated with ergosterol peroxide, ganodermanondiol, DMSO control, or serum stimulation (10%, 20% serum) controls as indicated. Treatment doses of a, b, c corresponded to 20, 10, and 5 μg/ml, respectively. The E2F-GFP reporter activities (A) were measured after 1 and 2 day(s) of treatment, with the E2F-OFF and -ON levels labelled alongside the serum starved (0.02%) and stimulated (20%) control samples, respectively. The Click-iT EdU assays (B) were performed after 1.5 days of treatment to detect subpopulations of cells that exited quiescence (with positive EdU incorporation) and divided (with incorporated EdU intensity reduced by half). The PI DNA staining assays (C) were performed after 1 day of treatment for cell cycle profile analysis. (D) Computer simulated percentages of cells that exit quiescence (y-axis) in response to serum input at indicated concentrations (x-axis). Quiescence exit was determined by the event of turning ON the Rb-E2F bistable switch in stochastic model simulations (see Methods) [21]. The serum concentration corresponding to 50% quiescence exit indicated the activation threshold of the Rb-E2F bistable switch. The parameter k0 corresponded to the rate constant of basal E2F expression. Arrow-pointed data points refer to percentages of quiescence-exit cells with k0 =0 and 0.06, respectively, at the 0.5% low serum condition. (E) Shallow vs. deep quiescence in compound-treated cells. Ergosterol peroxide and ganodermanondiol reduce the quiescence depth of treated cells, which are primed for quiescence exit and cell cycle re-entry compared to non-treated cells.

In serum starvation-induced quiescent REF/E23 cells, ergosterol peroxide and ganodermanondiol treatment led to increased E2F activity in a compound dose-dependent manner (Figure 3A). The REF/E23 cells contain a stably integrated E2F1 promoter-driven destabilized EGFP (E2F-GFP) reporter that has been previously calibrated with the endogenous E2F activity [21]. After 1 day of treatment, both ergosterol peroxide and ganodermanondiol caused an up to 2–3 fold increase of E2F-GFP reporter activity compared to DMSO control. After 2 days of treatment, ergosterol peroxide but not ganodermanondiol further increased E2F-GFP reporter activity to ~6 fold over DMSO control (20 μg/ml ergosterol peroxide, Figure 3A). The increased E2F activity resulted from ergosterol peroxide and ganodermanondiol treatment was still lower than the E2F activity in proliferating cells (with 20% serum, Figure 3A). Correspondingly, ergosterol peroxide and ganodermanondiol treated serum-starved REF/E23 cells mostly remained quiescent and did not enter the cell cycle, as indicated by their overall negative EdU incorporation (Figure 3B) and cell cycle profiles with non-replicated (2n) DNA content (Figure 3C).

Increased E2F activity in quiescent cells can drive cells to a shallow quiescent state. Computer simulations based on our previously established Rb-E2F bistable switch model [21] suggested that increasing basal E2F expression reduced the serum threshold to activate the Rb-E2F bitable switch (Figure 3D). For example, increasing the rate constant of basal E2F expression (*k_0_*) from 0 to 0.06 led to a significant increase (from 0 to 46%) in the percentage of quiescence-exit cells at a low serum (0.5%) condition (arrow-pointed, Figure 3D). Therefore, with increased E2F expression, quiescent cells become sensitized to weak growth signals and spontaneous quiescence exit (Figure 3E). Since quiescence provides a protection against cellular stress and toxicity [24, 25], the reduced quiescence depth likely sensitizes cells to cytotoxic effects of ergosterol peroxide and ganodermanondiol. Consistently, we noticed that compared to ganodermanondiol, ergosterol peroxide caused stronger cytotoxic and apoptotic effects (Figure 2A and Supplementary Figure 2A–2B) in quiescent REF/E23 cells and meanwhile a larger increase of E2F activity (Figure 3A and Supplementary Figure 2C) under both quiescent conditions of serum starvation and contact inhibition.

### Quiescent MCF7 breast cancer cells vs. MCF10A normal breast epithelial cells are more sensitive to ergosterol peroxide and ganodermanondiol treatment

**Figure 4:**
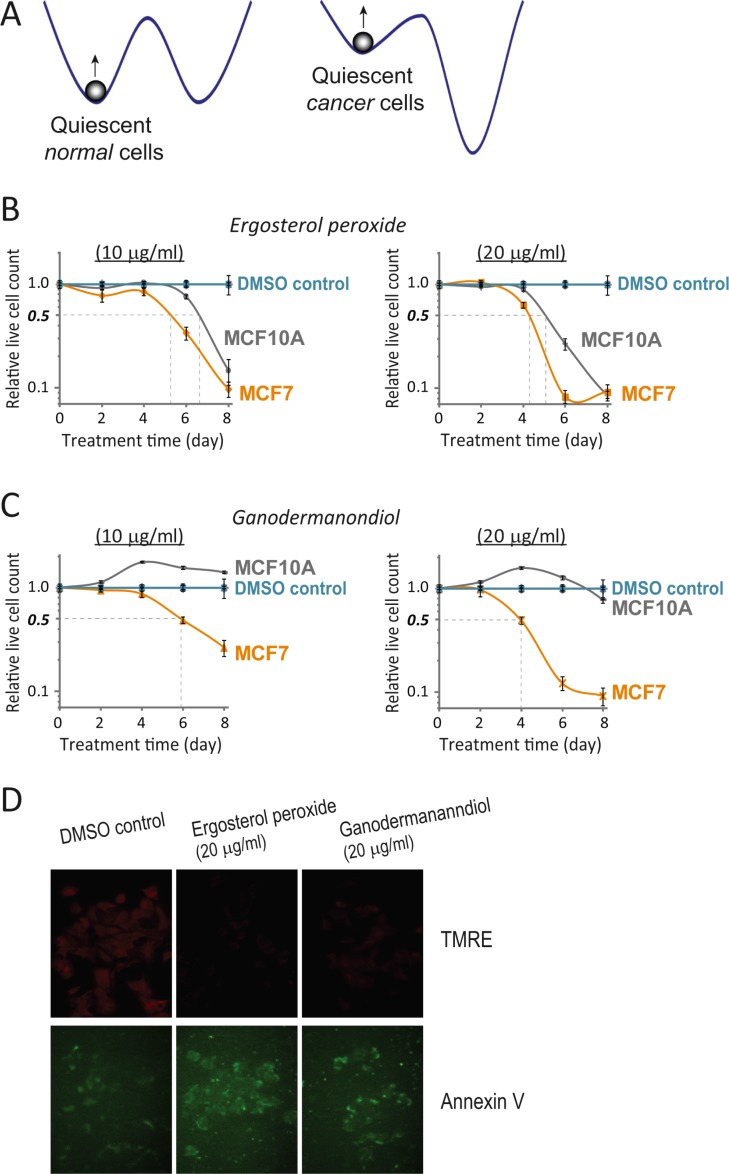
Quiescent MCF7 cells are more sensitive to compound cytotoxicity than quiescent MCF10A cells (A) Quiescent states in cancer versus normal cells. Cancer cells featuring autonomous cell proliferation presumably have a shallower and unstable quiescent state compared to normal cells. (B, C) Compound cytotoxicity time course in quiescent MCF7 vs. MCF10A cells. Serum starvation-induced quiescent MCF7 and MCF10A cells were treated with ergosterol peroxide (B) or ganodermanondiol (C) at 10 and 20 μg/ml as indicated. The relative live cell count (y-axis) was determined as in Figure 1B, at the indicated day (x-axis) during the course of treatment. Error bar indicates the standard error of the median (of four replicates). (D) Induced apoptosis in quiescent MCF7 cells. Serum starvation-induced quiescent MCF7 cells were treated with ergosterol peroxide or ganodermanondiol at 20 μg/ml or DMSO vehicle control. Cells were assayed for mitochondrial membrane potential (TMRE) and externalization of inner membrane phospholipids (Annexin V) after 2 days of treatment.

One of the hallmarks of cancer cells is the disrupted control of the cell cycle, which leads to autonomous cell proliferation in the absence of strong growth signals and despite of tight cell contact. Yet, the quiescent state is still present in many if not all cancer cells, so that in a population of fast proliferating cancer cells, a sub- or side-population can be quiescent or slow cycling, exhibiting stem cell-like characteristics [38–43]. Consistently, many transformed cancer cell lines (e.g., Hela, MCF7, U2OS) cease proliferation and enter quiescence-like state when deprived of serum growth signals. The quiescent state in cancer cells is presumably shallower and less stable compared to that in non-cancer cells (Figure 4A).

We reasoned that because of their relatively shallow quiescent state, cancer cells are easier to be pushed out of quiescence compared to non-cancer cells (Figure 4A). Therefore, by further reducing the quiescence depth, ergosterol peroxide and ganodermanondiol treatment is likely to sensitize cancer cells to cytotoxic effects, while leaving non-cancer cells less vulnerable. We compared MCF7 breast cancer cells and their non-transformed counterpart MCF10A breast epithelial cells in this regard. Both MCF7 and MCF10A cells were induced to quiescence by serum starvation for 2 days and then subject to ergosterol peroxide and ganodermanondiol treatment for up to 8 days. We found that indeed both compounds caused stronger cytotoxic effects in quiescent MCF7 vs. MCF10A cells. Specifically, with ergosterol peroxide treatment at two tested doses (10 and 20 μg/ml), the time to reach a 50% reduction in live cell count of quiescent MCF10A cells was ~1 day delayed than that of quiescent MCF7 cells (Figure 4B). The delayed cell killing effect in quiescent MCF10A vs. MCF7 cells was even more significant with ganodermanondiol – e.g., ganodermanondiol treatment at 20 μg/ml for 4 days led to a 50% reduction of MCF7 cell count but no reduction of MCF10A cell count; 4 days later (by day 8), the reduction of MCF7 cell count was over 90% but that of MCF10A was only ~20% (Figure 4C). Further test indicated that the cytotoxicity caused by ergosterol peroxide and ganodermanondiol in quiescent MCF7 cells was due to apoptosis induction, as shown by the corresponding loss of mitochondrial membrane potential and externalization of inner membrane phospholipids to cell surface (Figure 4D).

## DISCUSSION

In this study we tested and found that ergosterol peroxide and ganodermanondiol, two natural compounds purified from *Ganoderma lucidum*, exhibited cytotoxicity against not only proliferating cells but quiescent, slow-cycling cells by inducing apoptosis. We found that these two compounds increased the basal activity of the Rb-E2F bistable switch in quiescent cells, resulting in a reduction of quiescence depth and sensitization of cells to quiescence exit and cytotoxicity. Consistently, cancer cells (e.g., MCF7) at presumably less stable quiescent state compared to quiescent normal cells (e.g., MCF10A) were found preferentially killed by ergosterol peroxide and ganodermanondiol treatment.

The cytotoxic effect of natural *Ganoderma lucidum* compounds in targeting quiescent slow-cycling cells revealed an underappreciated mechanism of the well documented antitumor effects of *Ganoderma lucidum* active components, in addition to the immunomodulatory effects of polysaccharides and suppression of cell proliferation by triterpenoids [6]. The ability to target and eliminate quiescent slow-cycling cancer cells may also help the development of chemotherapeutic agents against cancer stem and progenitor cells, which is critical for the prevention of cancer recurrence.

Still, several significant questions remain unanswered. We do not know how the compound treatment increases E2F basal activity and how exactly the compound treatment induces apoptosis. Common chemotherapeutic drugs such as doxorubicin, paclitaxel and topotecan are known to induce apoptosis preferentially in actively cycling cells by damaging DNA or microtubules, or by inhibiting enzymes necessary for proliferation. Quiescent cells without active DNA replication and cell division are thus much less sensitive to these drugs (Figure 2C). The apoptotic effects of ergosterol peroxide and ganodermanondiol in quiescent cells are associated with the increase of E2F basal activity. The increased E2F activity serves as a marker for and meanwhile directly contributes to reducing quiescent depth (and thus reducing quiescence-associated protection against cytotoxicity). In addition, E2F overexpression itself is known to induce apoptosis [44, 45]. The increased E2F activity may therefore sensitize cells to apoptotic effects in addition to quiescence exit. Yet, increased E2F expression alone is likely not solely responsible for apoptosis induction in quiescent cells. In our tests, knocking down E2F1 expression in quiescent REF/E23 cells by siRNAs did not significantly block compound cytotoxicity (Dai et al., unpublished data), although it can be partially explained by the compensation among different E2F isoforms [46]. The sensitivity of a quiescent cell to compound-induced apoptosis is likely affected by both the E2F-dependent quiescence shallowing and sensitization effects, and cellular stress responses to quiescence signals (lack of serum growth/survival factors or high cell density) and compound cytotoxicity. Different cell types at different growth conditions (proliferation vs. quiescence) may exhibit varying sensitivity to compound induced cytotoxic and apoptotic effects. Lastly, ergosterol peroxide and ganodermanondiol are two out of hundreds or more of natural compounds contained in *Ganoderma lucidum*. Synergistic and/or antagonistic antitumor effects may exist among various active components of *Ganoderma lucidum*, awaiting future investigations.

## MATERIALS AND METHODS

### Natural compound purification from *Ganoderma lucidum*

Similar to our previously developed purification methods [19, 20], the fruiting body *Ganoderma lucidum* (Fujian Xianzhilou Biol. Sci. & Tech., China) was first crushed and extracted with ethanol. The extract was concentrated under reduced pressure until no ethanol smell remained, and subsequently extracted with petroleum ether to remove fat, followed by extraction with chloroform until the extract became colorless. The chloroform extract was concentrated under reduced pressure, and the concentrate was evaporated to dryness on a water bath to obtain *Ganoderma lucidum* triterpenoids. The obtained triterpenoid extracts were dissolved in chloroform, and then extracted several times with saturated aqueous NaHCO_3_. The basic aqueous layer in the extract was removed, and the remaining extract solution was washed with water until neutral. The extract was then again concentrated under reduced pressure and evaporated to dryness on a water bath to obtain neutral triterpenoids. The neutral triterpenoids were added to a silica gel column chromatography and subject to a petroleum ether–ethyl acetate–methanol gradient elution, giving rise to a total of 13 elution parts. The 4^th^ elution part was added to a silica gel column chromatography and further subject to a petroleum ether–ethyl acetate gradient elution, resulting in 22 fractions, among which the 16th fraction was identified as ergosterol peroxide. The 6th elution part was added to a silica gel column chromatography and further subject to a petroleum ether–ethyl acetate gradient elution, resulting in 10 fractions. The 6th fraction was dissolved in methanol, and subsequently purified using preparative high performance liquid chromatography (HPLC) to obtain ganodermanondiol.

### Cell lines and culture conditions

REF/E23 cells were derived from rat embryonic fibroblast REF52 cells as a single cell clone containing a stably integrated E2F1 promoter-driven destabilized EGFP reporter as previously described [21]. MCF7 and MCF10A cells were a gift from Dr. Schroeder (UA). REF/E23 cells were maintained in Dulbecco's Modified Eagle's Medium (DMEM) (No. 15–013, Corning, plus 2% GlutaMax, No. 35050, Gibco) supplemented with 10% bovine growth serum (BGS, No. SH3054103, HyClone, GE Healthcare). MCF7 cells were maintained in DMEM supplemented with 10% fetal bovine serum (FBS, No. SH3091003, HyClone, GE Healthcare). MCF10A cells were maintained in DMEM/F12 medium (No.10–090, Corning) supplemented with 5% horse serum (No. DH-05, Omega Scientific), 20 ng/ml epidermal growth factor (EGF, No. 354052, Corning), 0.5 μg/ml hydrocortisone (No. H0888, Sigma), 100 ng/ml cholera toxin (No. C8052, Sigma), and 10 μg/ml insulin (I6634, Sigma). HL-60 cells were maintained in RPMI medium with 10% FBS and 2% penicillin-streptomycin.

### Quiescence induction

Cells were induced to quiescence by serum starvation or cell contact inhibition. For serum starvation, growing cells were trypsinized and seeded at about 30–50% confluency in 6-well or 96-well cell culture plates, washed twice with DMEM after cell attachment, and cultured for 2 days in serum-starvation medium (for REF/E23 cells, 0.02% BGS in DMEM; for MCF7 cells, 0.02% FBS in DMEM without phenol red; for MCF10A cells, DMEM/F12 medium supplemented with 0.5 μg/ml hydrocortisone, 100 ng/ml cholera toxin, and 10 μg/ml insulin, without horse serum and EGF). For contact inhibition, growing REF/E23 cells were trypsinized and seeded at about 5 × 10^5^ cells/well (~200% confluency) in 6-well cell culture plates and subsequently cultured in 10% BGS for 3 days. Cells induced to quiescence were confirmed by their E2F-OFF state, 2n DNA profile, negative EdU incorporation (Figure 3A–3C) and the apparent lack of cell division seen under microscope.

### Compound and drug treatment

For treatment with natural *Ganoderma lucidum* compounds, ergosterol peroxide and ganodermanondiol were added to the culture medium to the final concentrations as indicated. For treatment with chemotherapy drugs, doxorubicin (44583, Sigma), paclitaxel (T1912, Sigma), and topotecan (T2705, Sigma) were added to the culture medium to the final concentrations as indicated. Cells were incubated with compounds/drugs or DMSO vehicle control for the indicated durations. In all cytotoxicity time course experiments, the compound-containing culture medium was changed every other day.

### Cytotoxicity and apoptosis assays

Cytotoxicity was determined at indicated time points by comparing the live cell count in compound-treated samples and DMSO control samples using the MTS assay (for HL-60 suspension cells) and propidium iodide (PI) fluorescence assay (for REF/E23, MCF7 and MCF10A adherent cells). The MTS assay was performed using the CellTiter 96 AQueous One Solution Cell Proliferation Assay kit according to the manufacturer's protocol (No. G3582, Promega). The PI fluorescence assay was performed as previously described [47], which exhibited a linear relationship (Supplementary Figure 4) between PI fluorescence intensity and live cell count (determined by deducting the number of non-vital cells assessed in the 1st PI fluorescence reading from the total cell number assessed in the 2nd reading after cell freezing and thawing). Apoptosis was assayed using the Multi-parameter Apoptosis Assay Kit according to the manufacturer's protocol (KA1335, Abnova), which includes TMRE, FITC-conjugated Annexin V, and Hoechst dye for the detection of mitochondrial membrane potential, phosphatidylserine on the outer membrane, and to demonstrate nuclear morphology, respectively.

### E2F activity and cell cycle assays

To measure E2F activity in individual cells, REF/E23 cells were harvested by trypsinization, fixed with 1% formaldehyde, and measured for the fluorescence intensity of the E2F-GFP reporter. For the EdU cell proliferation assay, EdU (1 μM) was added to culture medium with compound treatment. After 1.5 days of culturing with EdU, cells were trypsinized and subjected to a Click-iT EdU assay according to the manufacturer's protocol (No. C10419, Life Technologies, Thermo Fisher). For the cell cycle analysis, cells were trypsinized, spun down, and resuspended in Nuclear Isolation Medium (0.5% BSA and 0.1% NP-40 in PBS) with propidium iodide (PI, 5 μg/ml) and 1% RNase A (R5000, Sigma). Flow cytometry was used to measure GFP, EdU, and PI signal intensity in individual cells. Approximately 5,000–10,000 cells from each sample were measured using an LSR II flow cytometer (BD Biosciences), and were analyzed using FlowJo software (v. 10.0).

### Stochastic model simulation

We developed a stochastic differential equation (SDE) model based on our previously developed Rb-E2F bistable switch model (Supplementary Table 1) [21], with parameter units and species concentrations converted to molecule numbers. We adopted the chemical Langevin formulation [48, 49]:

$$X_{i}(t+\tau )=X_{i}(t)+\sum_{j=1}^{M}v_{ij}a_{j}[\text{X(t)}]\tau +\theta \sum_{j=1}^{M}v_{ij}{\left(a_{j}[X(t)]\tau \right)}^{1/2}\gamma +\delta \omega \tau^{1/2}$$

where *X_i_*(*t*) denotes the molecule number of species i (i = 1,…,n) at time *t*, and X(t) = (X1(t),…,Xn(t)) is the system state at *t*. *a*_j_ [*X (t)*] (j = 1,…,M) describes the temporal evolution of the system while *V_ji_* describes the molecule number change of i. Factors *γ* and *ω* are statistically independent temporally uncorrelated normal Gaussian noises, which are adjusted by scaling factors *θ* and *δ*, respectively ( *θ* = 0.35, *δ* = 30). The E2F-ON state in a given cell was determined by the molecule number of E2F over a cut-off value of 300 at the 24th model hour under a serum input. SDEs were implemented and solved in MATLAB.

## SUPPLEMENTARY MATERIALS FIGURES AND TABLES


