# Supplementary material for: Elimination of quiescent slow-cycling cells via reducing quiescence depth by natural compounds purified from Ganoderma lucidum
Source: Oncotarget. 2017 Jan 13;8(8):13770–81. doi: 10.18632/oncotarget.14634 (PMC5355137; doi:10.18632/oncotarget.14634)
Supplement: Supplementary file 1 [file oncotarget-08-13770-s001.pdf]

Elimination of quiescent slow-cycling cells via reducing quiescence depth by natural compounds purified from *Ganoderma lucidum*

Supplementary Materials

Supplementary Table 1: The Rb-E2F switch model (adapted from ref. [21])

$\frac{d[M]}{dt}=\frac{k_M[S]}{K_S+[S]}-d_M[M]$
$\frac{d[E]}{dt}=k_E\left(\frac{[\mathrm{M}]}{\mathrm{K_M}+[\mathrm{M}]}\right)\left(\frac{[E]}{K_E+[E]}\right)+\frac{k_b[M]}{K_M+[M]}+k_{E0}+\frac{k_{P1}[CD][RE]}{K_{CD}+[RE]}+\frac{k_{P2}[CE][RE]}{K_{CE}+[RE]}-d_E[E]-k_{RE}[R][E]$
$\frac{d[CD]}{dt}=\frac{k_{CD}[M]}{K_M+[M]}+\frac{k_{CDS}[S]}{K_S+[S]}-d_{CD}[CD]$
$\frac{d[CE]}{dt}=\frac{k_{CE}[E]}{K_E+[E]}-d_{CE}[CE]$
$\frac{d[R]}{dt}=k_R+\frac{k_{DP}[RP]}{K_{RP}+[RP]}-k_{RE}[R][E]-\frac{k_{P1}[CD][R]}{K_{CD}+[R]}-\frac{k_{P2}[CE][R]}{K_{CE}+[R]}-d_R[R]$
$\frac{d[RP]}{dt}=\frac{k_{P1}[CD][R]}{K_{CD}+[R]}+\frac{k_{P2}[CE][R]}{K_{CE}+[R]}+\frac{k_{P1}[CD][RE]}{K_{CD}+[RE]}+\frac{k_{P2}[CE][RE]}{K_{CE}+[RE]}-\frac{k_{DP}[RP]}{K_{RP}+[RP]}-d_{RP}[RP]$
$\frac{d[RE]}{dt}=k_{RE}[R][E]-\frac{k_{P1}[CD][RE]}{K_{CD}+[RE]}-\frac{k_{P2}[CE][RE]}{K_{CE}+[RE]}-d_{RE}[RE]$

Model variables:
S, [serum]; *M*, Myc; *E*, E2F; *CD*, Cyclin D/Cdk4,6; *CE*, Cyclin E/Cdk2;
R, Rb; *RP*,phosphorylated Rb; *RE*, Rb-E2F complex
Initial condition:
[M] = [E] = [CD] = [CE] = [RP] = 0 nM; [R] = 2.65 nM; [RE] = 0.65 nM.
Model parameters (see Supplementary Table 2).

Supplementary Table 2: Model parameters (adapted from ref. [21])

Symbol	Values	Description
k_M	1.0 nM/hr	Rate constant of Myc synthesis driven by growth factors
k_E	0.4 nM/hr	Rate constant of E2F synthesis driven by Myc and E2F
k_b	0.003 nM/hr	Rate constant of E2F synthesis driven by Myc alone
k_{E0}	0.02 nM/hr	Rate constant of E2F basal/constitutive synthesis
k_{CD}	0.03 nM/hr	Rate constant of CycD synthesis driven by Myc
k_{CDS}	0.45 nM/hr	Rate constant of CycD synthesis driven by growth factors
k_{CE}	0.35 nM/hr	Rate constant of CycE synthesis driven by E2F
k_R	0.18 nM/hr	Rate constant of Rb constitutive synthesis
k_{P1}	18 hr^{-1}	Phosphorylation rate constant of Rb by CycD/Cdk4,6
k_{P2}	18 hr^{-1}	Phosphorylation rate constant of Rb by CycE/Cdk2
k_{DP}	3.6 nM/hr	Dephosphorylation rate constant of Rb by phosphatases
k_{RE}	180 nM/hr	Association rate constant of Rb and E2F
K_S	2.8 nM	Michaelis-Menten parameter for CycD and Myc synthesis by growth factors
K_E	0.15 nM	Michaelis-Menten parameter for CycE and E2F synthesis by E2F
K_M	0.15 nM	Michaelis-Menten parameter for CycD and E2F synthesis by Myc
K_{RP}	0.01 nM	Michaelis-Menten parameter for Rb dephosphorylation
K_{CD}	0.92 nM	Michaelis-Menten parameter for Rb phosphorylation by CycD/Cdk4,6
K_{CE}	0.92 nM	Michaelis-Menten parameter for Rb phosphorylation by CycE/Cdk2
d_M	0.7 hr^{-1}	Degradation rate constant of Myc
d_E	0.25 hr^{-1}	Degradation rate constant of E2F
d_{CD}	1.5 hr^{-1}	Degradation rate constant of CycD
d_{CE}	1 hr^{-1}	Degradation rate constant of CycE
d_R	0.06 hr^{-1}	Degradation rate constant of Rb
d_{RP}	0.06 hr^{-1}	Degradation rate constant of phosphorylated Rb
d_{RE}	0.03 hr^{-1}	Degradation rate constant of Rb-E2F complex

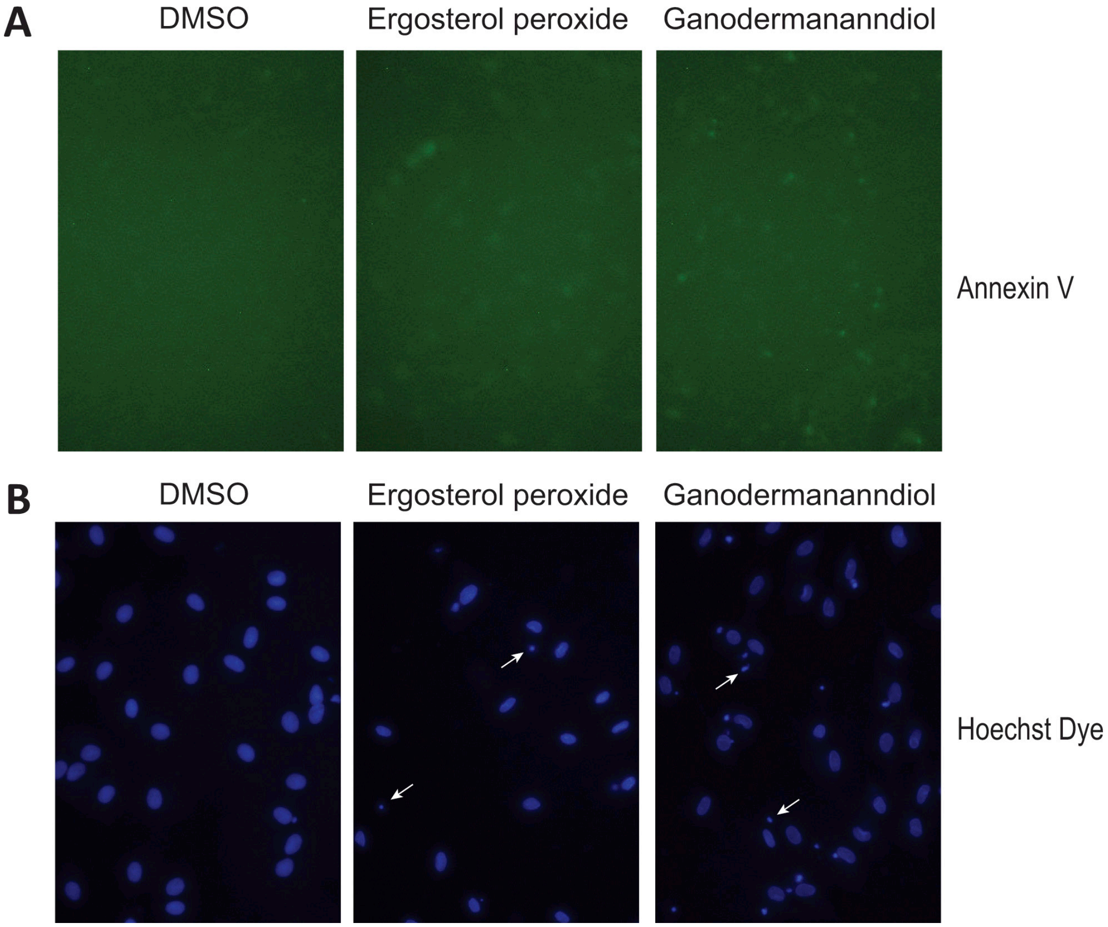

Supplementary Figure 1: Compound-induced apoptosis in REF/E23 cell-quiescence model. Serum starvation-induced quiescent REF/E23 cells were treated with ergosterol peroxide or ganodermanondiol at 20 µg/ml or DMSO vehicle control. After 1 day of treatment, cells were assayed for externalization of inner membrane phospholipids to cell surface using Annexin V staining (**A**), as well as nuclei condensation and fragmentation (examples shown at arrow-pointed spots) using Hoechst dye staining (**B**), in addition to the loss of mitochondrial membrane potential using TMRE staining (Figure 2B).

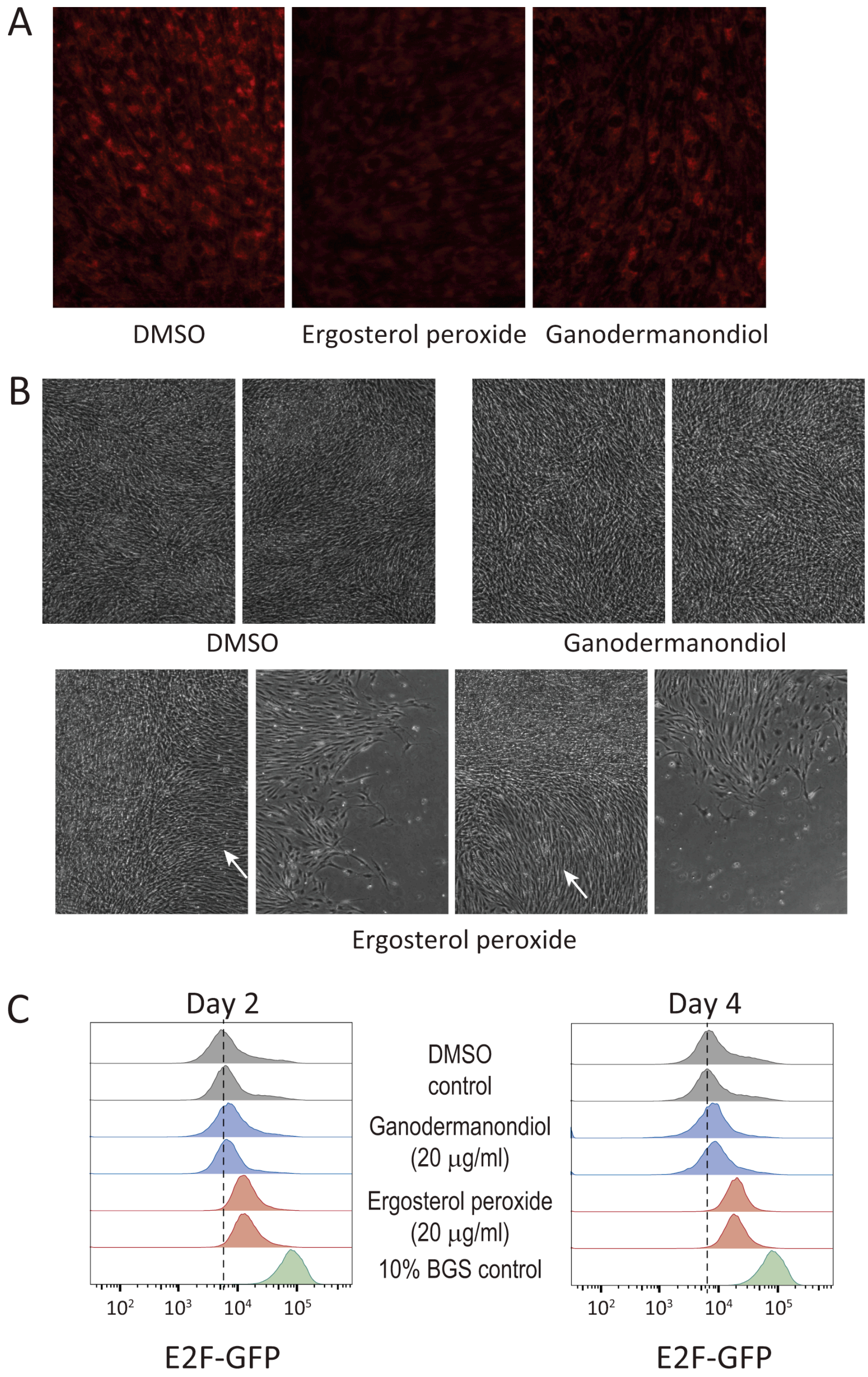

Supplementary Figure 2: Compound-induced cytotoxicity, apoptosis, and shallow quiescence under cell contact inhibition. REF/E23 cells were induced to quiescence by cell contact inhibition (see Methods) for 3 days, and subsequently treated with ergosterol peroxide or ganodermanondiol at 20 μg/ml or DMSO vehicle control. (**A**) Induced apoptosis. After 3 days of treatment, cells were assayed for the loss of mitochondrial membrane potential using TMRE staining. (**B**) Cytotoxicity. Cells were observed under microscope each day after the treatment initiation. At day 8, certain areas of ergosterol peroxide-treated wells (but not DMSO- or ganodermanondiol-treated wells) were seen to contain empty space or cells with significantly increased sizes (arrow-pointed). Shown are representative microscopy fields under phase contrast. (**C**) Shallower quiescent state with increased E2F expression. The E2F-GFP reporter activities were measured after 2 and 4 days of treatment by flow cytometry. The 10% BGS control refers to a population of cycling REF/E23 cells under 10% BGS without contact inhibition.

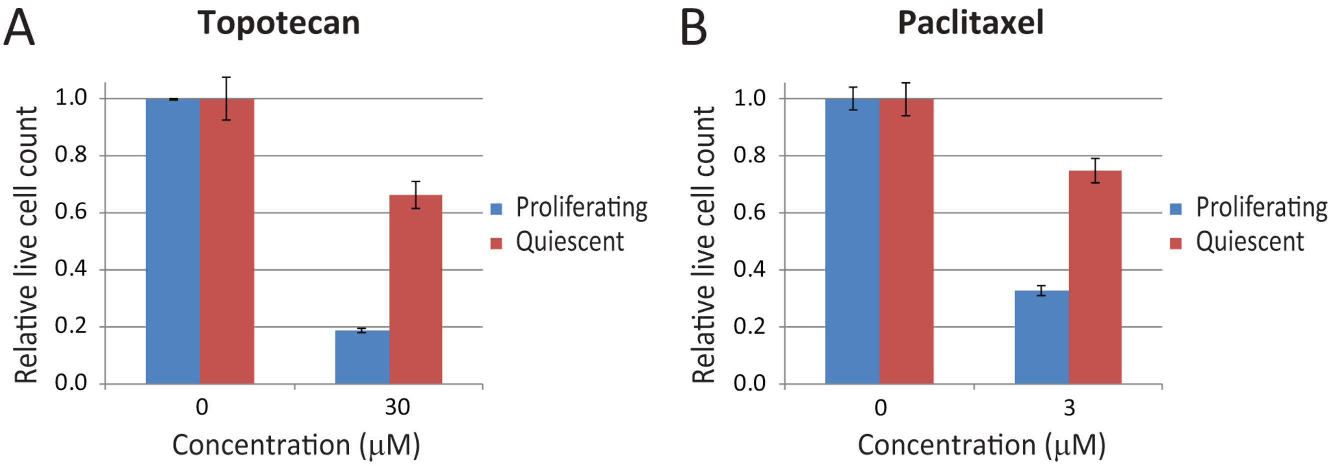

Supplementary Figure 3: Cytotoxicity of topotecan and paclitaxel at high dose. Actively cycling or serum starvation-induced quiescent REF/E23 cells were treated with topotecan (**A**) and paclitaxel (**B**) for 48 hours at a higher concentration than those in Figure 2C (concentration = 0: DMSO vehicle control). The relative live cell count (y-axis) was subsequently determined as in Figure 1B. Error bar indicates the standard error of the median (of four replicates).

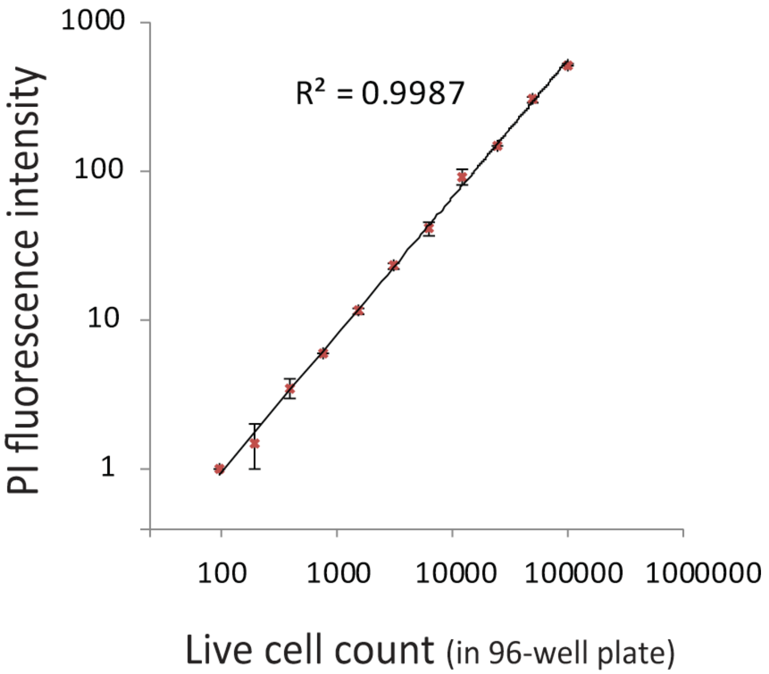

Supplementary Figure 4: Linear relationship between PI fluorescence intensity and live cell count in PI fluorescence assay. The PI fluorescence assay was performed as previously described [47], with 50 μg/ml of propidium iodide staining of REF/E23 cells. Each data point corresponds to the average reading from two replicates. Error bar indicates the standard error of the mean.
